# Blood Pressure Difference Between Cuff Inflation and Deflation by Auscultatory Method: Impact of Hypertension Grade

**DOI:** 10.3390/diagnostics15060687

**Published:** 2025-03-11

**Authors:** Francesca Coccina, Jacopo Pizzicannella, Oriana Trubiani, Sante D. Pierdomenico

**Affiliations:** 1 Department of Innovative Technologies in Medicine & Dentistry, University “Gabriele d’Annunzio”, Chieti-Pescara, 66100 Chieti, Italy; f.coccina@gmail.com (F.C.); oriana.trubiani@unich.it (O.T.); 2 Department of Engineering and Geology, University “Gabriele d’Annunzio”, Chieti-Pescara, 66100 Chieti, Italy; jacopo.pizzicannella@unich.it

**Keywords:** blood pressure, measurement, auscultatory method, hypertension

## Abstract

**Background**: The aim of this study was to evaluate blood pressure (BP) difference between cuff inflation and deflation and to investigate whether hypertension grade and patients’ characteristics could be influential. **Methods**: We selected 328 asymptomatic patients, 219 with mild systolic/diastolic hypertension, 75 with moderate systolic/diastolic hypertension and 34 with severe systolic/diastolic hypertension. **Results**: Hypertension grade was a determinant of the difference between systolic/diastolic BP during cuff inflation and deflation; the difference was progressively and significantly higher from mild to moderate to severe hypertension (1.80 ± 1.03/1.21 ± 0.56 vs. 5.32 ± 1.09/3.04 ± 0.81 vs. 9.74 ± 1.46/4.88 ± 0.73 mmHg, respectively (all differences were significant). Age, gender, body mass index, smoking habits and laboratory parameters were not associated with BP differences. The observed differences led to a reclassification of 24% of patients with moderate and 32% of patients with severe hypertension to a lower grade, but all were classified as hypertensive patients during cuff inflation. **Conclusions**: Hypertension grade influences the difference in systolic/diastolic BP during cuff inflation and deflation. This difference leads to a reclassification of hypertension grade during cuff inflation within the hypertensive range but does not influence the definition of hypertensive status. Future studies are needed to confirm whether the differences in systolic/diastolic BP between cuff inflation and deflation are due to BP increases induced by sympathetic activation, as well as a potential different behavior of the brachial artery during closing or opening, or both.

## 1. Introduction

Historically, the non-invasive measurement of blood pressure (BP) in the upper arm by the manual auscultatory method with a mercury or mercury-free sphygmomanometer has been the gold standard for hypertension diagnosis and management, as well as for risk stratification and cardiovascular disease prevention [1,2,3]. According to guidelines, the cuff should firstly be inflated to at least 30 mm Hg above the point at which the radial pulse disappears and then deflated at a rate of 2–3 mmHg/s [1,2]. Auscultation with a stethoscope positioned over the brachial artery in the antecubital fossa detects the appearance of two consecutive clear tapping sounds named Korotkoff phase I and denoting systolic BP, and the point at which all sounds disappear named Korotkoff phase V and designating diastolic BP [1,2]. However, Korotkoff sounds may also be heard during cuff inflation. Indeed, during cuff inflation, a first Korotkoff sound is heard, corresponding to the last Korotkoff sound detected during cuff deflation and intuitively identifying diastolic BP, and a last Korotkoff sound associated with the radial pulse disappearance is heard, corresponding to the first Korotkoff sound noticed during cuff deflation and intuitively identifying systolic BP. It can also be noticed that the BP value at which the first Korotkoff sound is heard during inflation and the last Korotkoff sound is heard during deflation, as well as the last Korotkoff sound during inflation and the first Korotkoff sound during deflation, may not be the same. This phenomenon has been described in some reports [4,5,6,7,8] using different methods and with differing results. The discrepancy between BP values recorded during cuff inflation and deflation by Korotkoff sound auscultation has been suggested to be related to a different vascular system behavior throughout arterial closing and opening [5,7]. However, it could also be related to cuff pressure-induced discomfort or pain, which may be proportional to the degree of BP reached in the cuff during inflation and which in turn may activate the sympathetic nervous system [9,10,11,12,13,14], further increasing BP values. In this context, this response could be primarily influenced by the degree of hypertension. Moreover, it could also be influenced by other factors influencing discomfort or pain [13,14,15,16,17], such as age, gender and additional patient characteristics, which in turn stimulate the sympathetic nervous system. At present, to the best of our knowledge, no study has evaluated whether the difference between BP values recorded during cuff inflation and deflation could be related to all the aforesaid factors.

The aim of this study was to evaluate the occurrence of differences in BP values recorded during cuff inflation and deflation and to investigate whether hypertension grade and other patients’ characteristics could influence this difference.

## 2. Materials and Methods

### 2.1. Patients

From the hypertensive population referred to our center for a diagnostic work-up, we selected 328 patients, that is, 219 patients with mild systolic and diastolic hypertension, 75 patients with moderate systolic and diastolic hypertension and 34 patients with severe systolic and diastolic hypertension, defined according to ESH guidelines [3]. The age range was 30–80 years. All the patients were asymptomatic. Most patients included in this study were evaluated between 2010 and 2018.

Patients under antihypertensive treatment (present or previous), or with diabetes or estimated glomerular filtration rate < 60 mL/min or previous cardiovascular events were excluded from this study.

All the subjects underwent clinical evaluation and routine laboratory and instrumental investigations. The study population came from the same geographical area (Chieti and Pescara, Abruzzo, Italy). In 1992, a prospective observational study project, without interventions beyond a routine clinical assessment, on the prognostic value of clinic BP, ambulatory BP and other risk markers in patients attending our clinic was proposed and approved by our Institutional Review Committee. For this specific manuscript, ethical approval was waived because the procedures were part of a normal clinical diagnostic procedure. Subjects gave written informed consent to be included in the study and for anonymous data processing.

### 2.2. Clinic BP Measurement

Clinic BP was recorded by a physician using a mercury sphygmomanometer (Welch Allyn Speidel & Keller, Jungingen, Germany) [18] and appropriate-sized cuffs. Measurements were performed three times in a quiet room, 2 min apart, after at least 5 min of rest, and the mean value was used as the BP for the visit. During cuff inflation, initially, rapidly up to 50 mmHg, and then at a rate of 2–3 mmHg/s, the first Korotkoff sound was recorded as diastolic BP and the last Korotkoff sound after disappearance of radial pulse as systolic BP. Then, the cuff was inflated to at least 30 mm Hg above the point at which the radial pulse was undetectable. During cuff deflation at a rate of 2–3 mmHg/s, the first Korotkoff sound (phase I) was recorded as systolic BP and the last Korotkoff sound (phase V) as diastolic BP. The possible presence of an auscultatory gap was checked [19,20,21]. During cuff deflation, the following hypertension categories were identified: (1) Grade 1 hypertension as systolic BP 140–159 mmHg and diastolic BP 90–99 mmHg; (2) Grade 2 hypertension as systolic BP 160–179 mmHg and diastolic BP 100–109 mmHg; (3) Grade 3 hypertension as systolic BP ≥ 180 mmHg and diastolic BP ≥ 110 mmHg [3]. Clinic heart rate (HR) was recorded by pulse palpation for 30 s before cuff inflation and for 30 s after cuff deflation.

### 2.3. Statistical Analysis

The data are reported as means ± standard deviation or numbers and percentages. Groups were compared using one-way analysis of variance followed by a post hoc test (Scheffé), chi-squared or Fisher’s exact test with Bonferroni’s correction, and t test, where appropriate. Linear regression analysis was also used where appropriate. Statistical significance was defined as *p* < 0.05. Analyses were made with the SPSS 21 software package (SPSS Inc. Chicago, IL, USA). Graphs were made with Microsoft Excel 10 (Microsoft Corporation, Redmond, WA, USA).

## 3. Results

The characteristics of the population studied are reported in Table 1. Sex distribution, body mass index, glucose and estimated glomerular filtration rate were substantially similar across the groups. Age, prevalence of smokers and low-density lipoprotein cholesterol levels tended to be higher in patients with severe hypertension but did not attain statistical significance.

Clinic BP values during cuff inflation and deflation are reported in Table 2. Clinic BP during cuff deflation was significantly different among the groups by definition. The same occurred for clinic BP during cuff inflation. Clinic HR was significantly higher in patients with severe hypertension after cuff deflation, but no significant difference was observed among the groups before cuff inflation.

The differences between systolic BP, diastolic BP and HR recorded during cuff deflation and inflation are reported in Table 3. The differences between systolic BP, diastolic BP and HR were significantly higher in patients with moderate hypertension than in those with mild hypertension, and in those with severe hypertension than in those with mild or moderate hypertension.

Age, gender, body mass index, smoking habit, glucose, low-density lipoprotein cholesterol and estimated glomerular filtration rate were not significantly associated with systolic or diastolic BP differences during cuff deflation and inflation.

**Figure 1:**
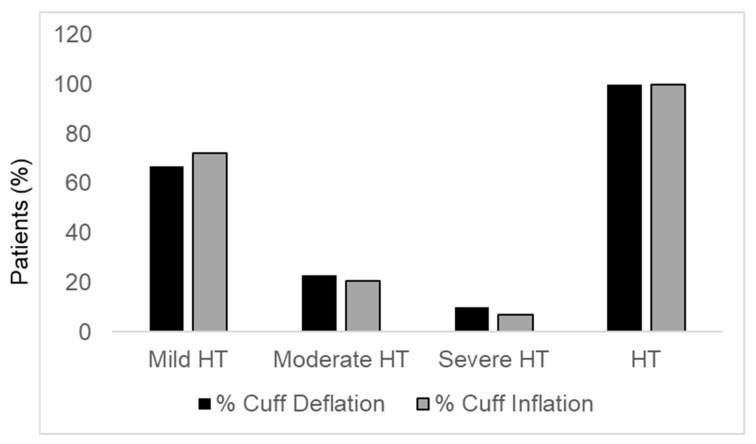
Prevalence mild hypertension, moderate hypertension, severe hypertension and hypertension itself during cuff deflation and cuff inflation. HT, hypertension.

Comparing systolic and/or diastolic BP values during cuff inflation with those during cuff deflation, no patient with mild hypertension was reclassified, 18 patients (24%) with moderate hypertension were reclassified as mild hypertensive patients, and 11 patients (32%) with severe hypertension were reclassified as moderate hypertensive patients. Globally, during cuff inflation 237, 68 and 23 patients were defined as having mild, moderate and severe hypertension, respectively. The global prevalence of hypertension grade and hypertension itself, based on systolic and/or diastolic BP, during cuff deflation and inflation is reported in Figure 1. The prevalence of mild hypertension was lower during cuff deflation than during cuff inflation (66.8 vs. 72.3%), whereas the prevalence of moderate and severe hypertension was higher during cuff deflation than during cuff inflation (22.9 vs. 20.7% and 10.4 vs. 7.0%, respectively). The prevalence of hypertension itself was similar between cuff deflation and cuff inflation (100 vs. 100%). The differences within each group between cuff deflation and cuff inflation, though numerically evident, did not attain statistical significance probably because of the small number of subjects in some groups. For the increase in mild hypertension, *p* = 0.12; for the decrease in moderate hypertension before and after inclusion of reclassified severe hypertensive patients, *p* = 0.08 and *p* = 0.51, respectively; and for the decrease in severe hypertension, *p* = 0.12.

## 4. Discussion

This study shows that hypertension grade influences the difference in systolic/diastolic BP during cuff inflation and deflation, being progressively higher from mild to moderate to severe hypertension. This difference leads to a reclassification of hypertension grade during cuff inflation to a lower grade in some patients with moderate and severe hypertension, but not in those with mild hypertension, and it does not influence the definition of hypertensive status.

It has been reported that during cuff inflation, sympathetic activation may occur, probably due to discomfort/pain induced by cuff pressure itself [9,10,11,12,13,14], which could further increase BP values. In such a context, the higher the cuff pressure, the greater the sympathetic reflex stimulation could be. Thus, our results documenting a progressive increase in the difference between BP detected during cuff inflation and deflation from mild to severe hypertension seem reasonably linked to the phenomenon mentioned above.

It has also been reported that discomfort/pain sensitivity, which may activate the sympathetic nervous system and further increase BP, may vary according to various factors [13,14,15,16,17]. For instance, it has been advocated that cuff pressure pain threshold may be lower in women than in men [13,14,15]. Moreover, it has been suggested that general pain threshold could increase with aging [16,17]. In our study population, gender and age were not significantly associated with BP difference between cuff inflation and deflation, suggesting that cuff pressure sensitivity during BP measurement was not influenced by these factors.

Some studies evaluated the difference between BP recorded during cuff inflation and deflation. Zheng et al. [4], with 15 healthy subjects, reported that manual systolic BP measured during cuff inflation was significantly lower by 4.3 mmHg than that measured during cuff deflation and that manual diastolic BP from cuff inflation was significantly higher by 3.0 mmHg than that from cuff deflation. The same authors—in another study [5], evaluating 40 normotensive subjects—showed that upon comparing BP values obtained during standard inflation with those from standard deflation, manual systolic BP was found to be 2.6 mmHg lower and manual diastolic BP was 1.5 mmHg higher (both statistically significant). Celler et al., when evaluating 15 subjects in 2023 [6] and assessing 40 sedated subjects with normotension/mild hypertension in 2024 [7], reported that systolic BP value recorded by the last Korotkoff sound during cuff inflation was underestimated by approximately 5 mmHg with respect to the systolic BP value recorded by the K1 Korotkoff phase during cuff deflation; in the same context, the estimation of diastolic BP during cuff inflation and deflation was similar [7]. In 2024 [8], Celler et al. also reported that systolic BP estimated from Korotkoff sounds during cuff inflation was on average 5 mmHg higher than during cuff deflation; in the same context, they reported that diastolic BP was on average 2.5 mmHg higher during cuff inflation than during cuff deflation. Regarding the difference between systolic BP during cuff inflation and deflation, our data are substantially in line with most previous findings, whereas concerning diastolic BP, some differences exist that could be related to population characteristics. Nevertheless, to the best of our knowledge, this is the first study to evaluate differences in systolic and diastolic BP during cuff inflation and deflation according to hypertension grade.

An aspect that deserves attention is to better clarify whether the differences in systolic/diastolic BP values recorded via the auscultatory method between cuff inflation and deflation are due to BP increases induced by discomfort- or pain-related sympathetic activation, which is proportional to cuff pressure during cuff inflation or to a potential different behavior of the brachial artery during closing and opening [5,7], which may influence the timing of Korotkoff sound auscultation, or both. This aspect warrants future investigations. In the case differences are related to BP increases during cuff inflation, according to our data, it could be hypothesized that particularly when severe hypertension is detected during cuff inflation, it may not be necessary to inflate the cuff at least 30 mm Hg above the point at which the radial pulse disappears, avoiding unnecessary discomfort/pain and additional BP increase.

In the context of BP measurement, it has been reported that brachial BP detected during cuff deflation tends to underestimate intra-arterial brachial systolic BP and overestimate intra-arterial brachial diastolic BP, and also shows small differences with intra-arterial aortic systolic BP [22,23]. Thus, according to our data, diastolic BP detected during cuff inflation could be closer to both intra-brachial and intra-aortic BP than diastolic BP detected during cuff deflation. Concerning systolic BP, future studies are needed to evaluate whether there are differences between systolic BP recorded during brachial cuff inflation and intra-aortic systolic BP.

This study has some limitations. First, we studied Caucasian subjects, and our results cannot be extrapolated to other ethnic groups. Second, due to the exclusion criteria, our data cannot be applied to treated hypertensive patients and those with diabetes, estimated glomerular filtration rate < 60 mL/min and previous cardiovascular events. Third, BP values were obtained by a single observer, and we did not have the possibility to perform an ideal study design including two separate readings performed by paired blinded observers. Fourth, we did not obtain a simultaneous intra-arterial radial, brachial or intra-aortic BP [7,22,23,24], or BP measured continuously and noninvasively by photoplethysmography [25,26], to evaluate whether the BP counterpart in these sites and with these methods differed significantly from diastolic and systolic BP values detected during cuff inflation by the auscultatory method. Nevertheless, it should be noted that there are differences in BP detected in these sites and with these methods [7,22,23,24,25,26]. Moreover, if this counterpart is not necessary during cuff deflation, it can be hypothesized that it may not be necessary even during cuff inflation.

## 5. Conclusions

Our data show that hypertension grade influences the difference in systolic/diastolic BP during cuff inflation and deflation, being progressively higher from mild to severe hypertension. This difference leads to a reclassification of hypertension grade during cuff inflation to a lower grade in some patients with moderate and severe hypertension, but not in those with mild hypertension, and it does not influence the definition of hypertensive status. Upcoming studies are needed to better clarify whether the differences in systolic/diastolic BP between cuff inflation and deflation are due to BP increase induced by sympathetic activation and also a potential different behavior of the brachial artery during closing or opening, or both. If it is confirmed in the future that the differences are mostly related to BP increase during cuff inflation and are proportional to cuff pressure itself, it could be hypothesized that when severe hypertension is detected during cuff inflation either by the appearance of the first Korotkoff sound (diastolic BP) or by the disappearance of the last Korotkoff sound and of the radial pulse (systolic BP), it may not be necessary to inflate the cuff at least 30 mm Hg above the point at which the radial pulse disappears, thereby avoiding unnecessary discomfort or pain and additional BP increase.

## Figures and Tables

**Table 1:** Characteristics of study groups.

Parameter	Mild HT	Moderate HT	Severe HT
*n*	219	75	34
Age, years	50 ± 11	52 ± 11	53 ± 9
Men, *n* (%)	107 (49)	39 (52)	17 (50)
Body mass index, kg/m^2^	26.5 ± 4	26.7 ± 4	27 ± 4
Smokers, *n* (%)	46 (21)	15 (20)	10 (29)
Glucose, mg/dL	91 ± 12	92 ± 12	92 ± 13
LDL cholesterol, mg/dL	130 ± 30	131 ± 34	135 ± 36
eGFR, mL/min	93 ± 14	93 ± 15	91 ± 14

eGFR, estimated glomerular filtration rate; HT, hypertension; LDL, low-density lipoprotein.

**Table 2:** Blood pressure and heart rate values of study groups.

Parameter	Mild HT	Moderate HT	Severe HT
Clinic SBP, mmHg (deflation)	148 ± 6	165 ± 6 *	185 ± 7 *†
Clinic DBP, mmHg (deflation)	94 ± 3	103 ± 3 *	113.5 ± 4 *†
Clinic SBP, mmHg (inflation)	146 ± 5	160 ± 5 *	175.5 ± 7 *†
Clinic DBP, mmHg (inflation)	93 ± 3	100 ± 3 *	108.5 ± 4 *†
Clinic HR, beats/min (after)	77 ± 11	77 ± 12	83 ± 10 ‡
Clinic HR, beats/min (before)	75 ± 11	72 ± 12	75 ± 10

DBP, diastolic blood pressure; HR, heart rate; HT, hypertension; SBP, systolic blood pressure. * *p* < 0.001 vs. mild HT; † *p* < 0.001 vs. moderate HT; ‡ *p* < 0.05 vs. mild and moderate HT.

**Table 3:** Difference between systolic blood pressure, diastolic blood pressure and heart rate recorded during cuff deflation and inflation in study groups.

Parameter	Mild HT	Moderate HT	Severe HT
Systolic BP, mmHg	1.80 ± 1.03	5.32 ± 1.09 *	9.74 ± 1.46 *†
Diastolic BP, mmHg	1.21 ± 0.56	3.04 ± 0.81 *	4.88 ± 0.73 *†
Heart rate, beats/min	1.58 ± 0.59	4.49 ± 0.93 *	7.79 ± 1.37 *†

Blood pressure data represent the blood pressure during cuff deflation minus the blood pressure during cuff inflation. Heart rate data represent the heart rate after cuff deflation minus the heart rate before cuff inflation. BP, blood pressure; HT, hypertension. * *p* < 0.001 vs. mild HT; † *p* < 0.001 vs. moderate HT.

## Data Availability

The data underlying this article are available on reasonable request from the corresponding author.
